# Systems analysis of the HPV–microbiome–biofilm triad

**DOI:** 10.3389/fcimb.2026.1767224

**Published:** 2026-03-17

**Authors:** Viktoria Nazarova, Nazira Kamzayeva, Talshyn Ukybassova, Samat Kozhakhmetov, Almagul Kushugulova

**Affiliations:** 1 Laboratory of Microbiome, Center for Life Sciences, National Laboratory Astana, Nazarbayev University, Astana, Kazakhstan; 2 Corporate Fund (CF) “University Medical Center”, Astana, Kazakhstan

**Keywords:** cervical cancer, cervicovaginal biofilms, co-infection, HPV, microbiome

## Abstract

**Background:**

Human papillomavirus (HPV) remains the leading cause of cervical cancer worldwide, however, its pathogenesis cannot be sufficiently explained by viral factors alone. Accumulating evidence highlights the critical role of cervicovaginal microbiome composition and biofilm formation in shaping viral persistence, epithelial barrier disruption and carcinogenic progression.

**Methods:**

This systems-based integrative synthesis analyzed peer-reviewed literature published between January 2000 and July 2025, retrieved from PubMed and Google Scholar with additional records identified through backward citation screening. The collected data were synthesized to construct a conceptual model of the HPV–microbiome–biofilm triad and to evaluate its clinical and biological implications.

**Results:**

The analysis indicates that depletion of Lactobacillus-dominated communities and expansion of anaerobic taxa, particularly Gardnerella vaginalis, are associated with biofilm development, chronic inflammation and immune modulation. These interrelated processes form self-reinforcing feedback loops that promote HPV persistence and reduce therapeutic efficacy. Microbiome dysbiosis and biofilm formation were further linked to impaired epithelial integrity, altered cytokine signaling pathways and clinically relevant phenotypes including immune escape, metabolic shifts and treatment non-responsiveness.

**Discussion:**

This systems perspective challenges reductionist pathogen-centered models and emphasizes the importance of integrating microbiome profiling and biofilm dynamics into cervical cancer risk stratification and therapeutic strategies. The coupled interactions between microbial communities, host immunity and viral persistence underscore the cervicovaginal ecosystem as an active regulator of disease progression rather than a passive bystander. Incorporating ecosystem-based parameters into clinical decision-making may enhance prognostic assessment and improve treatment outcomes, particularly in low- and middle-income countries where high HPV prevalence coincides with increased microbiome vulnerability.

**Systematic Review Registration:**

https://www.crd.york.ac.uk/PROSPERO/, identifier CRD420251208178.

## 1 Introduction

Human papillomavirus (HPV) is responsible for approximately 99.7% of cervical cancer cases worldwide and remains a major global public health challenge. Despite the widespread implementation of vaccination and screening programs, high-risk HPV types continue to drive cervical and oropharyngeal malignancies, with persistent infections progressing unpredictably to invasive disease. Current therapeutic strategies targeting individual pathogens show limited effectiveness in controlling persistent infections, highlighting fundamental gaps in our understanding of HPV pathogenesis (Molina et al., 2024).

Traditional models of HPV-associated carcinogenesis have primarily focused on viral factors alone. However, accumulating clinical evidence challenges this single-pathogen perspective. Multiple infections are common, occurring in more than half of patients, and often involve structured microbial consortia rather than random associations. These co-infections exhibit pronounced synergistic effects that cannot be explained by the simple additive impact of individual pathogens (Suehiro et al., 2019; Xie et al., 2021).

**Figure 1:**
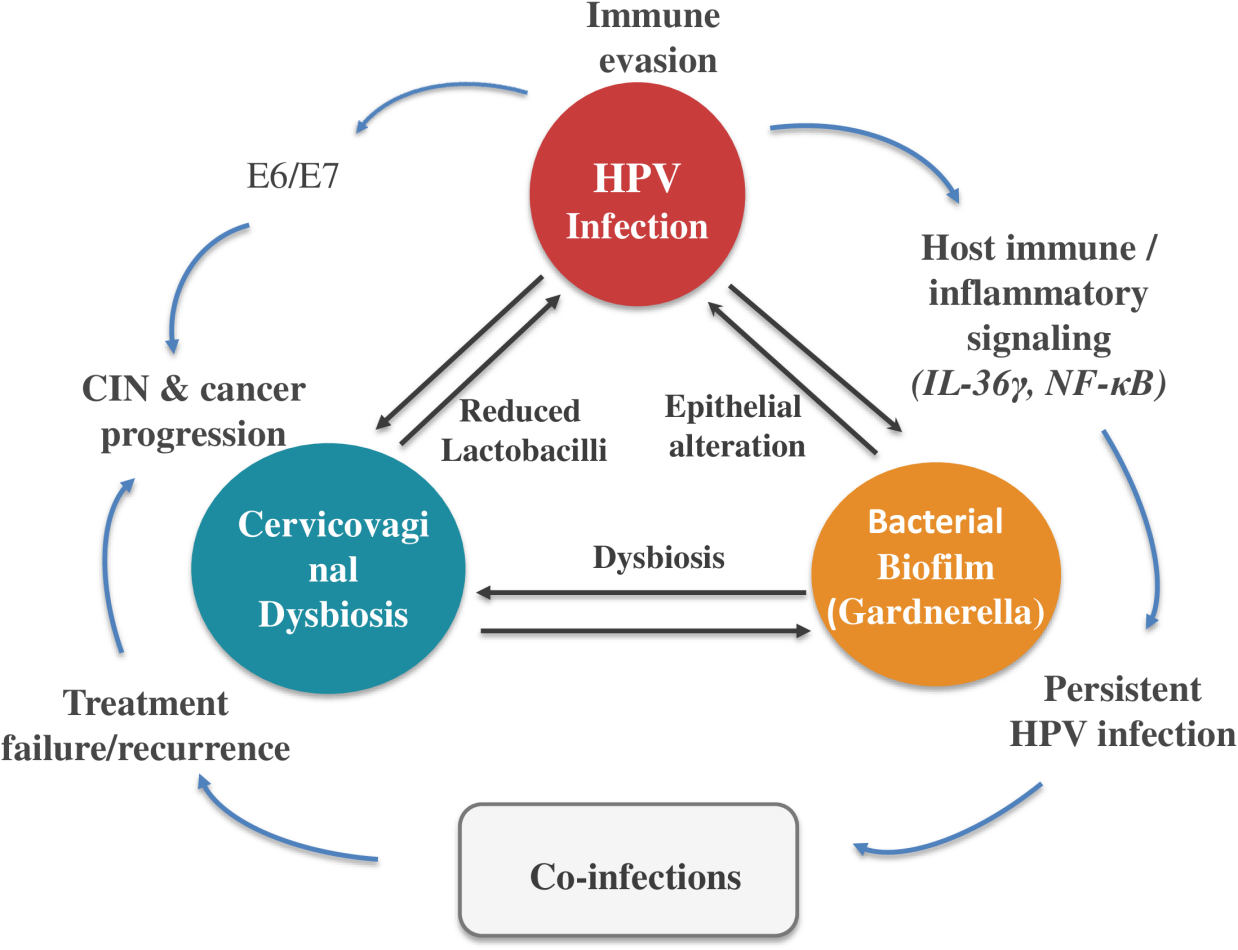
The HPV-microbiome-biofilm triad. Schematic representation of bidirectional interactions between HPV infection (including E6/E7-driven oncogenic activity), cervicovaginal dysbiosis, and bacterial biofilm formation (e.g., Gardnerella). These interconnected processes modulate host immune and inflammatory signaling (e.g., IL-36g, NF-kB), promoting HPV persistence, treatment failure/recurrence, and progression to cervical intraepithelial neoplasia and cancer. Co-infections are shown as additional modulators of the system.

We propose that HPV pathogenesis arises from complex interactions among viral, microbial, and biofilm components—an integrative framework referred to as the HPV–microbiome–biofilm triad (**Figure 1**). This model suggests that viral persistence and disease progression are driven not solely by viral attributes but by system-level effects arising from interactions among these components. Such dynamics may produce compensatory mechanisms that help explain the limited clinical efficacy of single-target therapeutic approaches (Jung et al., 2025).

The cervicovaginal microbiome displays distinctly divergent effects on HPV outcomes. Lactobacillus-dominated communities are associated with enhanced viral clearance, whereas dysbiotic anaerobic consortia create permissive environments that promote viral persistence (Dong et al., 2024). These interactions occur within complex biofilm architectures, where bacteria exist as matrix-encased communities demonstrating heightened antimicrobial resistance and creating microenvironments that may indirectly support viral persistence (Dong et al., 2023).

Co-infections further illustrate the complexity of this system. For example, *Chlamydia trachomatis* and HPV co-infection is associated with genomic instability that affects DNA repair processes and facilitates HPV integration. *C. trachomatis* disrupts host DNA repair pathways, while HPV exploits these compromised mechanisms to promote integration into the host genome (Wang et al., 2021). This coordinated subversion of cellular defense systems supports the persistence of both pathogens through interconnected inflammatory signaling pathways (Challagundla et al., 2023).

Understanding HPV pathogenesis as a complex interaction-driven network system carries important therapeutic implications (Ilhan et al., 2019). Conventional single-target therapies may inadvertently trigger compensatory responses in other system components, thereby limiting long-term effectiveness (Gottschick et al., 2017). Network-based interventions that simultaneously target multiple components while preventing adaptive switching may offer a paradigm shift toward more effective therapeutic strategies (Yang et al., 2024).

This systems analysis reveals how network-level properties contribute to adaptive resistance to current therapies and identifies potential intervention points for improving treatment outcomes in HPV-associated diseases. These insights hold the potential to transform clinical care for millions of patients with persistent HPV infections worldwide, particularly in resource-limited settings where the burden of HPV-related cancers is greatest. The goal of this systems analysis is to synthesize clinical and mechanistic evidence on the HPV–microbiome–biofilm triad to elucidate its impact on viral persistence, immune responses, and treatment resistance in cervical carcinogenesis.

## 2 Methods

### 2.1 Eligibility criteria

Studies were included if they were original peer-reviewed articles published in English between January 2000 and July 2025 and addressed the HPV–cervicovaginal microbiome–biofilm axis in the female lower genital tract. Eligible studies investigated HPV/hrHPV infection and/or HPV-associated cervical outcomes (e.g., HPV detection, viral load, persistence/clearance, cervical lesions/CIN, cervical cancer, post-treatment recurrence) in relation to cervicovaginal microbiota composition, bacterial vaginosis (BV)/dysbiosis, and/or biofilm-related mechanisms (BV-associated biofilms, *Gardnerella*, *Atopobium*, Lactobacillus depletion). Both clinical and experimental studies were eligible, including cross-sectional, case–control, and cohort designs, as well as *in vitro* laboratory-based investigations.

Non-original publications, including reviews, meta-analyses, editorials, commentaries, conference abstracts, and study protocols, were excluded during the initial screening stage prior to duplicate removal and title/abstract assessment.

At the full-text eligibility stage, studies were excluded if they focused on non-cervicovaginal anatomical sites or male-only populations; were not related to human papillomavirus (HPV) or high-risk HPV (hrHPV); did not assess cervicovaginal microbiota composition, bacterial vaginosis/dysbiosis, or biofilm-related features; or if the full text was unavailable. Reasons for exclusion at the full-text stage were documented and are summarized in the PRISMA 2020 flow diagram.

### 2.2 Search strategy

A systematic literature search was conducted in PubMed and Google Scholar to identify eligible studies published between January 2000 and July 2025. The final search was performed on 25 July 2025. Searches were limited to records with available abstracts and/or full-text access. In addition, backward citation searching (screening reference lists of included articles) was performed to identify additional relevant publications.

In PubMed, two Title/Abstract-based search queries were applied. The first query targeted studies addressing HPV in relation to the cervicovaginal microbiome and/or biofilms:

(HPV[Title/Abstract] OR “human papillomavirus”[Title/Abstract] OR hrHPV[Title/Abstract] OR “high-risk HPV”[Title/Abstract]) AND (vaginal[Title/Abstract] OR cervicovaginal[Title/Abstract] OR cervical[Title/Abstract] OR cervix[Title/Abstract]) AND (microbiome[Title/Abstract] OR microbiota[Title/Abstract] OR “bacterial vaginosis”[Title/Abstract] OR dysbiosis[Title/Abstract] OR biofilm*[Title/Abstract] OR Gardnerella[Title/Abstract] OR Lactobacillus[Title/Abstract]).

A second PubMed query focused on HPV co-infections:

(HPV[Title/Abstract] OR “human papillomavirus”[Title/Abstract] OR hrHPV[Title/Abstract]) AND (coinfection[Title/Abstract] OR co-infection[Title/Abstract] OR “co infection”[Title/Abstract]).

PubMed searches were restricted to 2000–July 2025, using Text availability filters (Abstract and Full text).

In Google Scholar, the following query was used to retrieve studies related to BV-associated biofilms: (“bacterial vaginosis” OR BV) AND (biofilm OR biofilms), restricted to 2000–2025, and the first 98 results were screened.

All retrieved records were imported into a screening dataset, duplicates were removed, and titles/abstracts were screened for relevance. Full texts were assessed against predefined eligibility criteria. Studies were included if they investigated HPV/hrHPV infection or HPV-associated cervical outcomes in relation to the cervicovaginal microbiota, bacterial vaginosis/dysbiosis, and/or biofilm-related mechanisms, including both clinical and *in vitro* experimental studies. Reasons for exclusion at the full-text stage were documented, and the study selection process is summarized in the PRISMA 2020 flow diagram.

### 2.3 Study selection process

All records identified through database searches (PubMed and Google Scholar) and citation tracking were imported into a reference management system, and duplicates were removed prior to screening. The study selection process followed the PRISMA 2020 guidelines.

In the first stage, titles and abstracts were independently screened for relevance based on the predefined inclusion and exclusion criteria. Studies clearly unrelated to HPV–cervicovaginal microbiome–biofilm interactions, focused on non-cervical HPV conditions or outside the defined study population and thematic scope were excluded.

Full-text articles deemed potentially eligible were subsequently retrieved and assessed in detail. Reasons for exclusion at the full-text stage were systematically documented and included: lack of relevance to cervicovaginal microbiome composition or biofilm formation, failure to meet methodological criteria, duplication or substantial overlap with previously included work, inaccessible full-text materials and inappropriate study design.

Clinical outcome definitions were extracted verbatim as reported in the original studies. In particular, HPV clearance was not uniformly defined across clinical studies and differed in terms of follow-up intervals and retesting criteria (e.g., HPV negativity at 6 vs 12 months). Due to this heterogeneity, clearance-related findings were synthesized qualitatively rather than pooled, and interpreted within the context of study-specific outcome definitions.

Any discrepancies arising during screening or full-text assessment were resolved through discussion and consensus. Studies meeting all predefined methodological and thematic requirements were included in the final systems analysis.

The complete selection pathway—including the number of records identified, screened, excluded and included—is presented in the PRISMA 2020 flow diagram (**Supplementary Figure B**: PRISMA 2020 Flowchart of Study Identification, screening and inclusion).

A total of 33 studies met the eligibility criteria. Nineteen observational studies collectively involved approximately 4,000 women of reproductive age from diverse geographic regions, while the remaining fourteen were experimental investigations employing cell-based and *in vitro* models. All eligible studies were incorporated into the systems-level synthesis.

This systems analysis was prospectively registered in PROSPERO (registration ID: CRD420251208178).

### 2.4 Data extraction and synthesis

Data from the included studies were extracted into a standardized evidence matrix, covering study type, study design, number and characteristics of participants, geographic location, HPV status and genotype, microbiome assessment methods, presence and characterization of biofilms, and reported clinical or mechanistic outcomes related to HPV persistence, clearance, epithelial disruption and immune modulation. Where reported, we also recorded whether clinical studies accounted for key potential confounders (e.g., age, smoking, sexual behavior, contraceptive use, and co-infections), including the use of adjusted analyses. For experimental studies, additional information regarding model type (*in vitro*), biological specimens and key mechanistic findings was recorded. Study findings were synthesized narratively to identify convergent patterns, emerging mechanisms and network-level interactions within the HPV–microbiome–biofilm triad. This approach enabled the identification of system-level properties that extend beyond isolated associations and supported the integrative interpretation of clinical and mechanistic evidence.

### 2.5 Risk of bias assessment

The 33 studies included in this systems analysis were stratified by study design and evaluated using validated, design-specific instruments to ensure a transparent assessment of methodological quality and risk of bias. Cross-sectional studies were assessed using the AXIS tool, which evaluates the clarity of study objectives, appropriateness of the study design, sampling methodology, measurement validity, and potential sources of selection and reporting bias. Longitudinal cohort studies were evaluated using the Newcastle–Ottawa Scale (NOS), with emphasis on participant selection, comparability between groups, and the reliability of outcome assessment and follow-up. Randomized controlled trials (RCTs) were appraised using the Cochrane Risk of Bias 2 (RoB 2) tool, which assesses bias arising from the randomization process, deviations from intended interventions, missing outcome data, outcome measurement, and selective reporting.

Experimental investigations employing *in vitro* models were assessed using a combination of RoB 2 and SciRAP (Science in Risk Assessment and Policy) tools. These instruments evaluated key domains including experimental design integrity, exposure characterization, reliability of outcome measurements, control of contamination and confounding factors, reporting transparency and potential conflicts of interest. Risk of bias assessment was performed independently by two reviewers. Discrepancies were resolved through discussion, and where necessary, a third reviewer was consulted to achieve consensus. Each study was assigned an overall qualitative classification of risk (low, moderate or high), which was integrated into the interpretation of findings to contextualize the strength and reliability of the evidence. A detailed risk of bias assessment is presented in the **Supplementary Material** (**Supplementary Tables S1**, **S4**) and informed the critical appraisal within the narrative synthesis, ensuring that the conclusions of this systems analysis reflect the methodological robustness and credibility of the included evidence. Based on the AXIS-based appraisal of the observational studies, overall methodological quality was judged as moderate, with some concerns primarily related to cross-sectional designs, convenience sampling, and limited representativeness. Most studies reported objectives and methods clearly and applied appropriate analytical approaches; however, these recurrent limitations may increase susceptibility to selection bias and reduce generalizability. The AXIS assessments are summarized in **Supplementary Table S1**. Longitudinal studies assessed using the Newcastle–Ottawa Scale (NOS) showed overall “some concerns” risk of bias, with consistently low risk in exposure ascertainment, outcome assessment, and follow-up duration. Some concerns were mainly related to the selection of non-exposed cohorts, comparability between groups, and completeness of follow-up. These assessments are summarized in **Table 1** and were considered when interpreting the longitudinal evidence on HPV–microbiome associations.

**Table 1:** Newcastle–Ottawa Scale (NOS) risk of bias assessment for included cohort studies.

First author, year	Representativeness of exposed cohort	Selection of non-exposed cohort	Ascertainment of exposure	Outcome not present at start	Comparability of cohorts	Assessment of outcome	Follow-up duration adequate	Completeness of follow-up	Overall risk of bias (NOS-based)
Shi, 2022	Low risk	Some concerns	Low risk	Low risk	Some concerns	Low risk	Low risk	Some concerns	Some concerns
Molina et al., 2024	Low risk	Some concerns	Low risk	Low risk	Some concerns	Low risk	Low risk	Some concerns	Some concerns

The risk of bias assessment using the Cochrane RoB 2 tool indicated low risk of bias in the included randomized trial (**Table 2**). Ou et al. (2019) showed low risk of bias across all RoB 2 domains.

The SciRAP-based assessment of the included *in vitro* studies indicated predominantly low risk across major methodological domains, including the description of test systems, exposure characterization, and outcome assessment procedures. The primary limitations related to replication and sample size, along with occasional issues involving incomplete reporting and potential confounding—patterns that are common in experimental models addressing complex biofilm and molecular interactions. (**Supplementary Table S4**)

### 2.6 Data synthesis approach

A qualitative systems synthesis approach was employed to integrate findings from observational, clinical, and experimental studies examining the HPV–microbiome–biofilm triad. Due to substantial heterogeneity in study designs, populations, outcome measures, and analytical methods, quantitative meta-analysis was not performed. Instead, evidence was systematically synthesized to identify convergent biological mechanisms and interaction patterns across molecular, microbial, and clinical domains. Study findings were interpreted through a systems-based framework, focusing on how viral factors, microbial dysbiosis, and biofilm formation interact to generate emergent properties such as immune evasion, epithelial barrier disruption, and therapeutic resistance.

Results were organized thematically according to core system components, including HPV persistence and clearance dynamics, cervicovaginal microbiome composition, biofilm architecture, immune modulation, and clinical treatment outcomes. Relationships among these domains were examined to highlight feedback loops, synergistic effects, and network-level behaviors that extend beyond isolated pathogenic mechanisms. This integrative synthesis enabled a holistic interpretation of the evidence, supporting the conceptualization of HPV-associated disease progression as a complex adaptive system rather than a single-pathogen process, and facilitated the identification of potential intervention points for future therapeutic strategies.

## 3 Results

### 3.1 Study selection

A total of 1,529 records were identified through database searches in PubMed (n = 1,418) and Google Scholar (n = 98), as well as through citation tracking (n = 13). After removal of 29 duplicates, 1,500 unique records remained and were screened based on titles and abstracts. Following this screening stage, 1,478 records were excluded as not relevant.

The remaining 181 full-text articles were assessed for eligibility. After full-text evaluation, 161 studies were excluded for predefined reasons, and 20 studies met the inclusion criteria from database searches. An additional 13 eligible studies were identified through reference list screening, resulting in a total of 33 studies included in the systems analysis. Of these, 19 were clinical investigations (including cross-sectional studies, cohort studies, and randomized controlled trials), while the remaining 14 were experimental or mechanistic studies. The complete study selection process is illustrated in the PRISMA 2020 flow diagram (**Supplementary Figure B**).

### 3.2 Characteristics of included studies

The main characteristics of the studies included in this systems analysis are summarized in **Supplementary Tables S5**, **S6**. A detailed overview of observational, cross-sectional, and comparative studies examining HPV–microbiome–metabolome interactions is provided in **Supplementary Table S5**, while **Supplementary Table S6** presents the characteristics of experimental and mechanistic studies investigating HPV–microbiome–host interactions. Overall, the evidence base comprised a heterogeneous body of observational, cohort, and interventional clinical studies, as well as experimental investigations, reflecting diverse geographic regions, study designs, and analytical approaches.

Clinical and observational studies predominantly focused on associations between high-risk HPV infection and alterations in the cervicovaginal microbiome, metabolic profiles, and inflammatory or immune signatures. Most studies enrolled women of reproductive age and used molecular techniques such as 16S ribosomal RNA (rRNA) gene sequencing, metagenomic profiling and metabolomics to characterize microbial community structure and function. A consistent pattern emerged across cohorts from Europe, Asia, Africa and the Americas, demonstrating that HPV persistence and cervical disease severity are associated with depletion of Lactobacillus-dominated communities and enrichment of anaerobic taxa, including Gardnerella, Atopobium, Prevotella, Sneathia and Megasphaera. Several studies also highlighted the role of community state types (CSTs), particularly CST IV, in promoting viral persistence and dysbiosis-linked inflammation. Longitudinal cohort studies provided temporal insights, indicating that microbiome instability and dominance of BV-associated bacteria were predictive of persistent HPV infection, whereas stable Lactobacillus crispatus–dominated profiles were associated with viral clearance. Randomized controlled trials and prospective clinical studies evaluating probiotic or microbiome-modulating interventions showed mixed results, with no consistent improvement in HPV clearance but evidence of improved cytological outcomes and partial restoration of protective microbial profiles, underscoring the potential but yet unproven therapeutic relevance of microbiome-targeted strategies (**Supplementary Table S5**).

Experimental and mechanistic studies complemented clinical findings by elucidating how microbial biofilms, co-infections and metabolic alterations contribute to epithelial disruption, immune modulation, and environments favorable for HPV persistence and carcinogenesis. Together, these studies provide a multidimensional perspective on the HPV–microbiome–biofilm triad, underscoring its relevance to cervical disease progression and its potential as a target for integrated preventive and therapeutic interventions (**Supplementary Table S6**).

Across all included studies, risk-of-bias patterns varied according to study design. Cross-sectional studies demonstrated a moderate level of bias based on AXIS evaluations, largely reflecting limitations in sampling strategies and study design (**Supplementary Table S1**). *In vitro* experimental studies showed predominantly low risk of bias according to the SciRAP frameworks, indicating strong methodological control and reproducibility (**Supplementary Table S4**). Cohort studies assessed using the NOS tool were judged to have moderate to high methodological quality (**Table 1**). Randomized controlled trials included in the review were evaluated using the Cochrane RoB 2 tool (**Table 2**), which indicated an overall low risk of bias across domains. These quality assessments were integrated into the interpretation of the synthesized findings to ensure that conclusions reflect the strength and limitations of the available evidence.

**Table 2:** Cochrane RoB 2 assessment for included randomized trials.

Study	Randomization process	Deviations from intended interventions	Missing outcome data	Measurement of outcomes	Selection of reported results	Overall risk of bias
Ou et al., 2019	Low risk	Low risk	Low risk	Low risk	Low risk	Low risk

### 3.3 Main findings

#### 3.3.1 HPV integration mechanisms and cellular disruption

HPV employs an integration process that differs from that of many other DNA viruses. Because HPV lacks specialized integration enzymes, integration events tend to occur at chromosomal sites where host DNA repair pathways are already engaged or overwhelmed. Experimental evidence indicates that the viral oncoproteins E6 and E7 disrupt cellular DNA damage–response mechanisms, creating conditions that facilitate integration while inhibiting apoptosis in infected cells (Spardy et al., 2009). Mechanistic studies further show that E6 compromises p53 tumor suppressor activity, whereas E7 interferes with cell-cycle regulation, collectively promoting genomic instability that favors HPV integration (Longworth and Laimins, 2004; Fu et al., 2010).

#### 3.3.2 Microbiome–HPV interactions

Multiple cohort studies indicate that cervicovaginal microbiome composition is associated with HPV-related outcomes. Lactobacillus-dominated communities are consistently linked to higher rates of viral clearance and typically exhibit acidic pH, lower inflammatory signaling and more favorable antiviral immune profiles (Srinivasan et al., 2015; Łaniewski et al., 2018; Chen et al., 2019). In contrast, dysbiotic communities enriched with anaerobic taxa such as *Gardnerella*, *Prevotella*, *Sneathia* and *Atopobium* are associated with HPV persistence and are characterized by elevated pH, chronic inflammatory signatures and evidence of local immune modulation. Longitudinal studies support temporal associations in both directions: dysbiotic community states have been reported to precede and predict persistent hrHPV infection in some cohorts, whereas HPV acquisition and persistence may also be followed by shifts toward greater community instability and anaerobe enrichment over time (Lebeau et al., 2022). Geographic variation has been reported with some African populations exhibiting higher baseline dysbiosis prevalence, which may contribute to increased HPV persistence.

#### 3.3.3 Biofilm architecture and protective function

Biofilm-associated communities have been reported in HPV-related cervicovaginal dysbiosis. Multiple studies document highly structured three-dimensional microbial communities with channel networks and stratified microenvironments. Gardnerella often functions as a keystone organism, forming structural scaffolds that promote colonization by other bacteria through metabolic cooperation. Dual-species biofilm models demonstrate enhanced colonization when Gardnerella scaffolds are present, generating polymicrobial biofilms with complex metabolic gradients (Castro et al., 2019). Biofilm-associated bacteria exhibit dramatically increased antimicrobial tolerance compared with planktonic cells (Castro et al., 2017). Biofilm matrices may reduce antimicrobial penetration and thereby contribute to reduced treatment responsiveness in biofilm-associated dysbiosis. Biofilm development further alters bacterial gene expression, increasing production of virulence factors that disrupt epithelial barrier integrity (Castro et al., 2020).

#### 3.3.4 Synergistic effects of coinfections

Coinfections were associated with amplified pathogenic effects in several studies. The interaction between *Chlamydia trachomatis* and HPV is among the best characterized: *C. trachomatis* induces DNA damage while suppressing repair pathways, creating conditions that may facilitate HPV integration (Ferrera et al., 2023). HPV persistence in this context has been associated with enhanced genomic incorporation and accelerated progression in experimental and clinical observations (Bellaminutti et al., 2014). Similar interactions have been described for other coinfections. *Neisseria gonorrhoeae* has been linked to epithelial barrier disruption that may facilitate viral access, while certain herpesviruses have been reported to exhibit genomic or functional interactions with HPV that intensify carcinogenic potential (Garland et al., 2001; Samarawickrema et al., 2015).

#### 3.3.5 Metabolic alterations and system reorganization

Metabolomic investigations included in this review describe stage-associated shifts in metabolic diversity across HPV disease progression. Early infection and dysplasia have been associated with reduced metabolic diversity (Karim et al., 2013). In contrast, invasive cancer has been linked to increased metabolic diversity consistent with metabolic heterogeneity in advanced disease (Ilhan et al., 2019). Functional profiling studies report enrichment of dysbiosis-associated pathways related to mucin degradation, epithelial adhesion, and immune evasion, which may contribute to HPV persistence (Srinivasan et al., 2015). Additional studies suggest reciprocal metabolic interactions between dysbiotic communities and infected or transformed cells, including lactate accumulation and altered substrate utilization patterns that may support viral persistence and integration (Gottschick et al., 2017; Yang et al., 2024).

#### 3.3.6 Barrier disruption and immune impairment

Evidence across included studies indicates convergent viral and microbial contributions to epithelial barrier disruption and immune dysfunction. HPV oncoproteins have been reported to compromise epithelial junction integrity, potentially facilitating bacterial penetration. Microbiome-derived factors may further intensify barrier impairment, as shown in studies using bacterial culture supernatants (Wang et al., 2021). Immune alterations include suppression of interferon signaling in HPV-positive states and chronic, dysbiosis-associated inflammation. Biofilm matrices may additionally reduce immune recognition and limit therapeutic penetration, contributing to persistence of both bacterial communities and viral infection (Castro et al., 2017).

#### 3.3.7 Clinical validation and therapeutic implications

Clinical studies corroborate many mechanistic insights. Microbiome composition at the time of HPV diagnosis predicts viral persistence and treatment outcomes (Shi et al., 2022). Surgical and ablative therapies show reduced efficacy in women with biofilm-positive bacterial infections, likely due to the protective properties of biofilm architecture (Li et al., 2020; Rosca et al., 2022). Probiotic interventions demonstrate limited effectiveness when biofilms are established; sequential strategies involving biofilm disruption followed by targeted antimicrobials and probiotic restoration show greater promise (Gottschick et al., 2017; Ou et al., 2019; Sabbatini et al., 2020). Incorporating microbiome profiling into risk stratification improves predictive accuracy compared with traditional clinical parameters (Morales et al., 2022).

## 4 Discussion

### 4.1 Beyond single-pathogen models: clinical evidence for microbial interactions

Clinical evidence increasingly challenges the long-standing view of HPV as an isolated pathogen. Across multiple cohorts, women with dysbiotic cervicovaginal microbiomes consistently exhibit lower rates of spontaneous HPV clearance and reduced responsiveness to standard therapies, a pattern observed across diverse regions and populations (Alaoui Sosse et al., 2023). These findings suggest that HPV outcomes are shaped not only by viral factors, but also by the surrounding cervicovaginal ecosystem, including microbial community structure and biofilm-associated states.

Importantly, most human studies remain cross-sectional, which limits causal inference. However, available longitudinal evidence supports temporal associations in both directions: dysbiotic community states may increase the likelihood of subsequent HPV persistence, while HPV acquisition and persistent hrHPV infection may also be followed by shifts toward greater community instability and anaerobe enrichment. Together, these observations are consistent with reinforcing feedback dynamics between HPV persistence and dysbiosis.

The clinical implications are substantial. Surgical interventions—particularly loop electrosurgical excision procedures and cold knife conization—show reduced success in women with biofilm-positive bacterial communities, and recurrence rates are higher when dysbiosis is present (Rosca et al., 2022). Biofilm formation appears to be a key contributor to this reduced efficacy. These three-dimensional polymicrobial structures create protected ecological niches that limit therapeutic penetration and increase antimicrobial tolerance, correlating with treatment failure across multiple modalities (Li et al., 2020).

Across diverse methodologies, the included evidence supports consistent patterns of interaction among viral, microbial and biofilm components. Mechanistic findings align with clinical observations showing more aggressive disease in the presence of multiple infections (Rosca et al., 2022), while metabolic signatures correlate with transcriptional and functional changes, supporting cross-level coupling between microbial community states and host responses (Castro et al., 2017). Collectively, these findings highlight the limitations of single-pathogen frameworks and support the need for integrated models that incorporate microbiome and biofilm dynamics into HPV risk assessment and management (Alaoui Sosse et al., 2023).

### 4.2 Practical implications for clinical management

These findings have direct relevance for clinical decision-making. Microbiome assessment at the time of HPV diagnosis may enhance treatment selection and patient counseling. Women with Lactobacillus-dominant microbial communities demonstrate higher rates of spontaneous clearance and may be suitable for conservative management or extended observation. In contrast, individuals with anaerobe-dominant dysbiosis may require earlier or more aggressive interventions (Chen et al., 2019).

Evidence further suggests that addressing bacterial vaginosis before initiating HPV-directed treatment may improve outcomes; however, antibiotic monotherapy is often insufficient given the protective architecture of biofilms. In BV-focused randomized clinical evidence, metronidazole followed by a biofilm-disrupting amphoteric tenside pessary (WO3191) was evaluated as a sequential approach and reduced biofilm detection, although recurrence was not prevented (Gottschick et al., 2017). Sequential therapeutic strategies combining biofilm-targeted therapy, targeted antimicrobials and subsequent microbiome restoration remain biologically plausible; however, direct RCT evidence demonstrating improved HPV-related outcomes remains limited (Ou et al., 2019).

Integration of microbiome-informed biomarkers into cervical screening programs could meaningfully enhance risk stratification. Point-of-care assays detecting key dysbiosis-associated taxa may help identify patients who would benefit from intensified monitoring or adjunctive therapies.

### 4.3 Global health implications

Microbiome-informed approaches carry particular importance for global health. Low- and middle-income countries bear a disproportionate burden of cervical cancer and show higher prevalence of cervicovaginal dysbiosis, creating a synergistic “double burden” that elevates the risk of HPV persistence and treatment failure (Łaniewski et al., 2018).

Microbiome-targeted strategies hold potential as scalable, cost-effective tools to improve prevention and early intervention efforts. Economic modeling suggests that incorporating microbiome assessment into routine screening could reduce unnecessary procedures while increasing detection of high-risk cases, remaining within acceptable cost-effectiveness thresholds across healthcare systems. In resource-limited settings, low-cost interventions—such as probiotic supplementation, nutritional modification, or simplified biofilm-targeted regimens—may provide feasible benefits, though population-specific validation remains essential due to geographic microbiome variability.

### 4.4 Comparison with existing literature

Previous reviews have examined individual components of HPV pathogenesis—such as viral oncogene activity, shifts in cervical microbiota or biofilm-mediated antimicrobial resistance—but none have integrated these elements into a unified systems-level framework. Earlier microbiome-focused reviews have emphasized associations between cervical dysbiosis and HPV persistence, but have not incorporated biofilm architecture or mechanistic pathways linking microbial metabolism to viral integration. Similarly, prior HPV-centered analyses have treated microbial dysbiosis as a secondary phenomenon rather than an active driver of viral behavior. Compared with these earlier works the present systems analysis demonstrates that viral, microbial and biofilm components interact synergistically, contributing to clinically relevant phenotypes—such as immune evasion, metabolic adaptation and reduced treatment responsiveness— that are not explained when these components are studied in isolation. This integrated perspective expands upon previous literature by identifying common mechanistic pathways and highlighting the necessity of multi-target therapeutic approaches.

### 4.5 Therapeutic development opportunities

Emerging evidence supports therapeutic strategies targeting viral persistence, bacterial dysbiosis and biofilm architecture simultaneously. Sequential regimens involving biofilm-disrupting agents, targeted antimicrobials and probiotic restoration warrant rigorous clinical testing. Personalized treatment algorithms based on individual microbiome profiles may further optimize outcomes; machine-learning approaches could potentially improve risk prediction by integrating microbial and clinical variables, although robust development and external validation are needed. Importantly, model interpretability and assessment of transferability across geographically distinct populations will be essential before clinical implementation. Additionally, immunomodulatory therapies addressing inflammation associated with dysbiosis may promote viral clearance.

### 4.6 Economic considerations

Formal health-economic evaluations were not reported in the included studies. However, microbiome-informed risk stratification could potentially improve clinical decision-making by better identifying individuals at increased risk of HPV persistence and high-grade lesions, thereby reducing unnecessary procedures. Although microbiome-based diagnostics may be less resource-intensive than some existing testing approaches, their implementation—particularly in low-resource settings—will depend on the availability of reliable point-of-care platforms. Importantly, translation into routine practice will require investment in workforce training, standardized quality assurance, regulatory approval and sustainable reimbursement pathways.

### 4.7 Strengths and limitations

This systems analysis integrates clinical, mechanistic, and ecological evidence, offering a comprehensive perspective on HPV pathogenesis. The use of validated risk-of-bias instruments enhances methodological rigor. Nonetheless, several limitations warrant consideration. Many included human studies were cross-sectional, limiting causal inference. Heterogeneity in sequencing platforms, analytical pipelines, and sampling methods reduces comparability across cohorts. Geographic and population-level factors may further contribute to variability in cervicovaginal microbiome composition and HPV-related outcomes. Therefore, clinical evidence was synthesized qualitatively by region (Africa, Europe, Asia and the Americas). Although key patterns—such as reduced Lactobacillus dominance and increased anaerobic diversity—were broadly consistent across settings, differences in study populations and laboratory methodologies (including sequencing platforms and taxonomic resolution) may partially explain variation in reported taxa and effect sizes linked to HPV persistence or clearance, limiting cross-study comparability and generalizability. In addition, HPV clearance definitions were not standardized across studies (e.g., HPV negativity at 6 vs 12 months), limiting direct cross-study comparisons. Moreover, adjustment for important confounding factors (e.g., sexual behavior, smoking, and co-infections) was inconsistently reported across clinical studies, which may influence observed associations and limits causal interpretation. Additionally, *in vitro* and *in vivo* models cannot fully replicate the complexity of the human cervicovaginal environment. Moreover, reporting of experimental sample size as well as the number of biological and technical replicates was not consistently detailed across studies, which may limit reproducibility and should be considered when interpreting mechanistic findings. Despite these limitations, the consistency of findings across diverse methodologies supports the robustness of the system-level conclusions.

### 4.8 Clinical recommendations

From a clinical perspective, the available evidence supports consideration of cervicovaginal microbiome status as a contextual modifier of HPV-related outcomes. Where clinically appropriate, evaluation and management of bacterial vaginosis prior to HPV-directed interventions may be beneficial; however, current support is derived primarily from observational and mechanistic studies, and definitive clinical recommendations await confirmation in well-designed randomized trials. In cases of recurrent disease or suboptimal treatment response, biofilm-associated microbial states may represent a contributing factor. While standardized biofilm-disrupting regimens have not yet been validated in HPV-focused clinical trials, emerging data suggest that combined or sequential approaches targeting both microbial dysbiosis and biofilm architecture warrant further investigation. Continued integration of microbiome profiling into clinical research frameworks will be critical to inform future risk stratification and therapeutic strategies, particularly in settings with a high burden of HPV-related disease.

### 4.9 Future research directions

Future research should prioritize the development of integrated therapeutic and diagnostic approaches that address the interconnected roles of HPV, microbial dysbiosis, and biofilm formation in cervical carcinogenesis. Several areas require immediate investigation. First, clinical trials are needed to evaluate combination therapies that simultaneously target viral replication, cervicovaginal dysbiosis and biofilm structures. Such multimodal interventions have shown promise in preliminary studies but require rigorous validation. Second, microbiome-derived biomarkers must be developed and validated to improve risk stratification and prediction of treatment response. These biomarkers could support personalized management strategies and enhance early identification of high-risk patients.

From a translational perspective, further work is required to determine the cost-effectiveness of microbiome-informed cervical screening strategies and to develop point-of-care assays capable of detecting key bacterial taxa and biofilm-associated markers. In parallel, clinical development efforts should focus on establishing standardized protocols for microbiome assessment within HPV management, creating evidence-based guidelines for biofilm disruption prior to surgical or ablative procedures and implementing training programs for clinicians to improve understanding of microbiome–virus interactions. Finally, dedicated implementation frameworks are needed to support the integration of microbiome-informed approaches into healthcare systems, especially in resource-limited settings where the burden of cervical cancer is disproportionately high.

## 5 Conclusion

HPV-driven cervical carcinogenesis is classically explained by the activity of the viral oncoproteins E6/E7, which disrupt cell-cycle control, impair DNA damage responses and promote immune evasion. Our triad model does not replace this canonical framework but rather situates it within a broader ecological context by highlighting how cervicovaginal dysbiosis and biofilm formation may sustain inflammation and epithelial barrier dysfunction, thereby creating conditions that favor viral persistence and disease progression. Within this network, Gardnerella-dominant biofilms and host immune/inflammatory signaling emerge as key synergistic nodes that intersect with E6/E7-mediated oncogenic programs. Collectively, the available evidence supports cautious integration of microbiome-informed concepts into risk stratification and management, particularly for high-risk or treatment-refractory cases, while acknowledging that prospective, controlled studies are still needed to establish causality and optimize intervention strategies.

## Data Availability

Publicly available datasets were analyzed in this study. This data can be found here: This study is a systematic review. All data analyzed are available within the original published articles cited in the manuscript. No standalone dataset or repository is associated with this review. Therefore, no accession numbers or repository links apply.
