# Supplementary figures and images for: Systems analysis of the HPV–microbiome–biofilm triad
Source: Front Cell Infect Microbiol. 2026 Mar 17;16:1767224. doi: 10.3389/fcimb.2026.1767224 (PMC13036498; doi:10.3389/fcimb.2026.1767224)

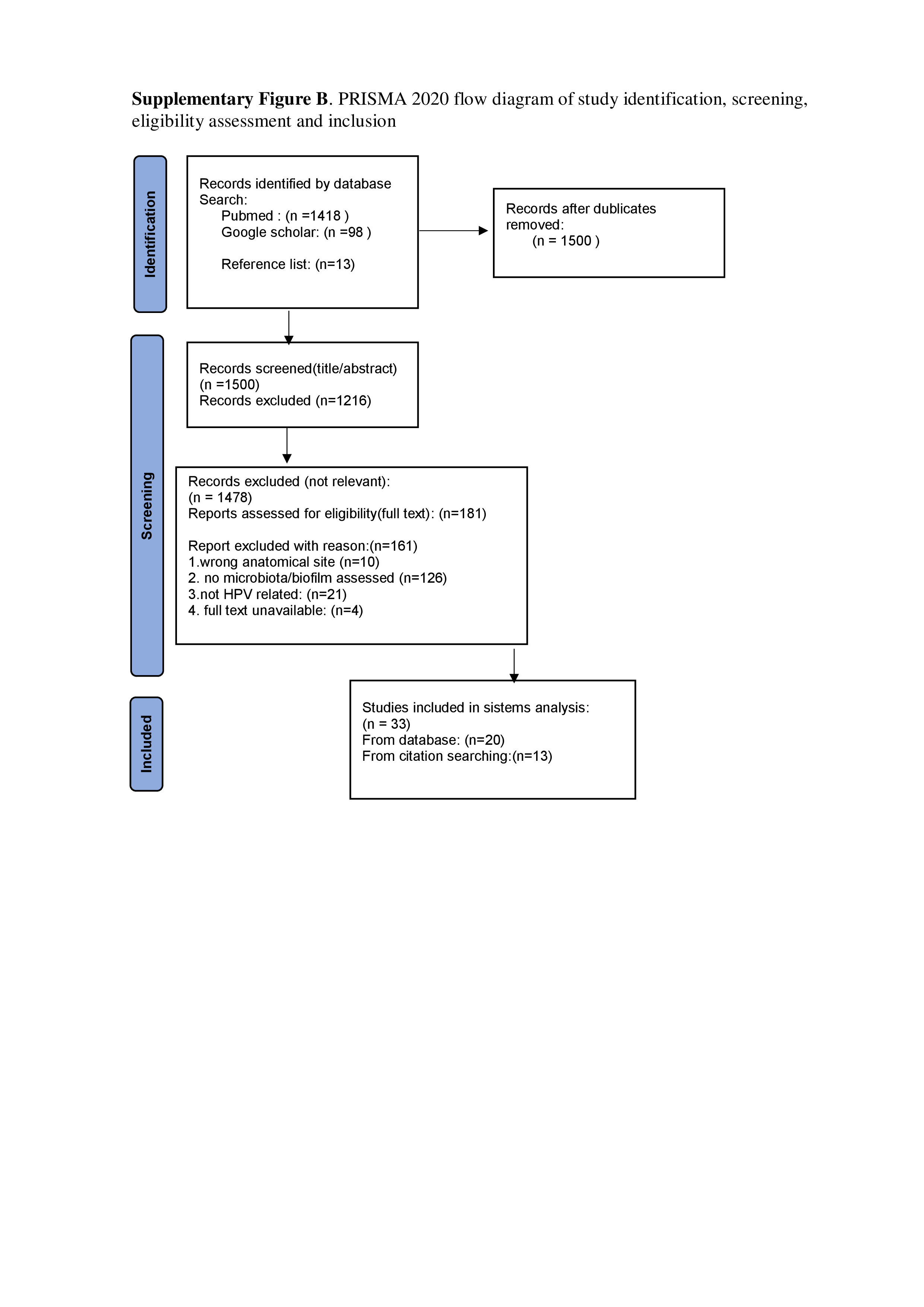

Supplement: Supplementary file 1 [file Image1.tiff]
