# Supplementary material for: Systems analysis of the HPV–microbiome–biofilm triad
Source: Front Cell Infect Microbiol. 2026 Mar 17;16:1767224. doi: 10.3389/fcimb.2026.1767224 (PMC13036498; doi:10.3389/fcimb.2026.1767224)
Supplement: Supplementary file 2 [file Table1.docx]

**Supplementary Table S1. AXIS-based quality assessment for observational studies**

| **First author, year of publication** | **Clarity of reporting** | **Study design** | **Data collection and sample selection** | **Internal consistency of results** | **Recognition of limitations** | **No conflicts of interest** | **Overall quality** |
| --- | --- | --- | --- | --- | --- | --- | --- |
| Ferrera L, 2023 | High | Cross-sectional | Moderate | High | Moderate | High | Moderate |
| Suehiro T.T., 2019 | High | Cross-sectional | Moderate | High | Moderate | High | Moderate |
| Xie et al., 2021 | Moderate | Retrospective cross-sectional | Moderate | High | Moderate | Moderate | Moderate |
| Jung D.R., 2025 | High | cross-sectional/metagenomic profiling | High | High | Moderate | High. | High |
| Dong, Y.H., 2024 | High | Cross-sectional observational | High | High | Moderate | High | High |
| Ilhan et al., 2019 | High | Observational cross-sectional with metabolomics integration | Moderate | High | Moderate | High | Moderate |
| Yang, 2024 | High | cross-sectional design | Moderate | Moderate | Moderate | High | Moderate |
| Bellaminutti, 2014 | High | Cross-sectional observational design | Moderate | High | Moderate | High | Moderate |
| Samarawickrema, 2015 | High | Cross-sectional clinic-based prevalence study | Moderate | High | High | High. | Moderate |
| Garland, 2001 | High | Cross-sectional clinic-based prevalence study | Moderate | High | Moderate | High. | Moderate |
| Morales et al., 2022 | High | Cross-sectional | Moderate | High | High | High. | Moderate |
| Alaoui Sosse et al., 2023 | Moderate | Cross-sectional observational study | Moderate | High | Moderate | Moderate | Moderate |
| Srinivasan et al., 2015 | High | Cross-sectional analytical case–control study | Moderate | High | Moderate | High | Moderate |
| Chen et al., 2019 | High | Cross-sectional observational study | Moderate | High | Moderate | High | Moderate |
| Łaniewski et al., 2018 | High | Cross-sectional observational comparative study | Moderate | High | Moderate | High | Moderate |
| Gottschick, 2017 | Moderate | Non-randomized prospective clinical study | High | Moderate | Moderate | Moderate | Moderate |
| Lebeau et al., 2022 | High | Cross-sectional | Moderate | High | Moderate | High | Moderate |
