# Supplementary material for: Systems analysis of the HPV–microbiome–biofilm triad
Source: Front Cell Infect Microbiol. 2026 Mar 17;16:1767224. doi: 10.3389/fcimb.2026.1767224 (PMC13036498; doi:10.3389/fcimb.2026.1767224)
Supplement: Supplementary file 3 [file Table4.docx]

**Supplementary Table S4. SciRAP-based reliability assessment for included in vitro experimental studies**

| **First author, year** | **Test system description** | **Exposure characterization** | **Study design clarity** | **Replication & sample size** | **Outcome assessment methods** | **Data completeness & reporting** | **Selective reporting** | **Contamination & confounding** | **No conflicts of interest** | **Funding bias** | **Overall reliability** |
| --- | --- | --- | --- | --- | --- | --- | --- | --- | --- | --- | --- |
| Fu, 2010 | Low risk | Low risk | Low risk | Moderate risk | Low risk | Moderate risk | Low risk | Low risk | Low risk | Low risk | Moderate risk |
| Longworth, 2004 | Low risk | Low risk | Low risk | Moderate risk | Low risk | Moderate risk | Low risk | Low risk | Low risk | Low risk | Moderate risk |
| Castro, 2019 | Low risk | Low risk | Low risk. | Moderate risk | Low risk | Moderate risk | Moderate risk | Moderate risk | Low risk | Low risk | Moderate risk |
| Castro, 2017 | Low risk | Low risk | Low risk | Moderate risk | Low risk | Low risk | Low risk | Low risk | Low risk | Low risk | Low risk |
| Castro, 2020 | Low risk | Low risk | Low risk | Moderate risk | Low risk | Low risk | Low risk | Moderate risk | Low risk | Low risk | Low risk |
| Karim, 2013 | Low risk | Low risk | Low risk | Moderate risk | Low risk | Low risk | Low risk | Low risk | Low risk | Low risk | Low risk |
| Li, 2020 | Low risk | Low risk | Low risk | Moderate risk | Low risk | Low risk | Low risk | Low risk | Low risk | Low risk | Low risk |
| Rosca, 2022 | Low risk | Low risk | Low risk | Low risk | Low risk | Low risk | Low risk | Low risk | Low risk | Low risk | Low risk |
| Sabbatini, 2020 | Low risk | Low risk | Low risk | Moderate risk | Low risk | Low risk | Low risk | Moderate risk | Low risk | Low risk k | Moderate risk |
| Spardy et al., 2009 | Low risk | Low risk | Low risk | Moderate | Low risk | Moderate risk | Low risk | Low risk | Low risk | Low risk | Moderate risk |
| Wang, 2021 | Low risk | Low risk | Low risk | Moderate risk | Low risk | Moderate risk | Low risk | Low risk | Low risk | Low risk | Low risk |
| Dong et al., 2023 | Low risk | Low risk | Low risk | Moderate risk | Low risk | Low risk | Moderate risk | Moderate risk | Low risk | Low risk | Low risk |
| Challagundla, 2023 | Low risk | Low risk | Low risk | Moderate risk | Low risk | Moderate risk | Low risk | Low risk | Low risk | Low risk | Moderate risk |
