# Supplementary material for: Systems analysis of the HPV–microbiome–biofilm triad
Source: Front Cell Infect Microbiol. 2026 Mar 17;16:1767224. doi: 10.3389/fcimb.2026.1767224 (PMC13036498; doi:10.3389/fcimb.2026.1767224)
Supplement: Supplementary file 4 [file Table5.docx]

**Supplementary Table S5. Summary of observational, cross-sectional, and comparative studies on HPV–microbiome–metabolome interactions**

| **№** | **Study (author, year)** | **Country** | **Design** | **Population (n, age, sex)** | **Intervention / Exposure** | **Comparator:** (HPV outcome) | **Outcomes measured** | **Key findings** |
| --- | --- | --- | --- | --- | --- | --- | --- | --- |
| 1 | Molina et al., 2024 | Netherlands | Longitudinal observational study | 141 hrHPV DNA-positive women | Temporal dynamics of cervicovaginal microbiome via metagenomic sequencing | Non-progression (NILM at 6 months) vs progression (LSIL/HSIL at 6 months) | Microbiome composition, diversity, and stability indices; HPV outcome | Persistent hrHPV infections associated with unstable, Gardnerella-dominant microbiomes; clearance correlated with sustained Lactobacillus-dominated states. Microbiome temporal 53patterns predict HPV outcomes. 24 months |
| **2** | **Xie et al., 2021** | China | Cross-sectional study | 668 women screened for HPV and STDs | Ten sexually transmitted pathogens (e.g. Chlamydia trachomatis, Ureaplasma urealyticum) and high-risk HPV co-infection | hrHPV+ vs hrHPV | Association between HPV and co-infecting pathogens | Demonstrates significant association between high-risk HPV and several STD pathogens, suggesting co-infection as a persistence factor |
| **3.** | Suehiro T.T. et al., 2019 | Brazil | Cross-sectional | 213 women, 18–72 years, female | Exposure: Human papillomavirus (HPV) infection and bacterial vaginosis (BV) | Normal cytology vs abnormal cytology (LSIL/HSIL ± cervical cancer) | High-grade squamous intraepithelial lesions (SIL), cervical cytology abnormalities | Prevalence of HPV-DNA: 69.9%; BV: 72.7%; Co-infection with BV and high-risk HPV associated with increased risk of SIL; Gardnerella vaginalis most common BV agent; co-infections may act as cofactors in HPV-mediated cervical lesions |
| **4** | **Jung et al., 2025** | South Korea | Metagenomic cross-sectional study | 68 women | Vaginal microbiome composition in HPV-positive women | **HPV16/18-positive women vs HPV-negative controls** | Taxonomic and functional differences in cervicovaginal microbiota | Reveals distinct microbial community profiles in HPV 16/18 infections, with reduced Lactobacillus and enrichment of anaerobes linked to persistence and dysbiosis |
| **5** | **Dong et al., 2024** | China | Comparative metagenomic study | 102 women | HPV infection and microbial community composition of urogenital tract and rectum | HPV-positive women vs HPV-negative women | Microbial diversity and inter-site microbial network changes | Shows HPV infection alters microbial structure and connectivity in both urogenital and rectal sites, suggesting systemic microbiome shifts |
| **9** | **Ilhan et al., 2019** | USA | Observational metabolomic study | 78 women (HPV-positive, HPV-negative, and cervical dysplasia cases) | Cervicovaginal microbiota and metabolic profiling | HPV− controls vs HPV+ controls vs cervical lesion severity groups (LSIL/HSIL/ICC) | Microbiome composition, inflammation, metabolic pathways | Reveals that HPV infection and dysbiosis alter cervicovaginal metabolic signatures, linking microbial metabolites, inflammation, and carcinogenesis |
| **10** | Gottschick et al., 2017 | Germany | Prospective clinical study with microbiome analysis | Included women (*n* = 44), 32.4 years (range 19–51 years) | Vaginal pessary containing amphoteric tenside (dequalinium compound) | Pre- vs post-treatment; BV vs healthy controls | BV recurrence rate, Nugent score, vaginal microbiota composition (16S rRNA sequencing) | Amphoteric tenside pessary significantly reduced BV symptoms and biofilm-associated bacteria (Gardnerella, Atopobium). Lactobacillus abundance increased post-treatment, suggesting microbiota restoration. |
| 11 | Yang et al., 2024 | China | Cross-sectional metabolomic and microbiome study | n=26 Women with and without high-risk HPV infection (36.37 ± 12.59 years) | High-risk HPV infection and cervicovaginal microbiota composition | hrHPV-positive vs hrHPV-negative women | Vaginal microbial and metabolic profiles; correlation between HPV and metabolite shifts | HR-HPV infection significantly associated with reduced Lactobacillus abundance and increased anaerobes (Gardnerella, Prevotella, Atopobium). Metabolomic shifts indicated altered amino acid and lipid pathways. Suggests HPV–microbiome–metabolome crosstalk in cervical microenvironment. |
| 15 | Srinivasan S et al., 2015 | USA | Observational cohort (metabolomic profiling) | 60 women with and without bacterial vaginosis | Vaginal metabolome analysis (LC–MS) | BV-positive vs BV-negative women | Metabolite profiles associated with BV | Identified metabolic biomarkers distinguishing BV; findings provide insights into metabolic pathways linked to dysbiosis relevant for HPV-related pathogenesis. |
| 16 | Chen et al., 2019 | China | Cross-sectional observational study | n = 135 women (pregnant and nonpregnant, reproductive age); all female | High-risk HPV (hrHPV) infection and pregnancy status; analysis of vaginal microbiome by 16S rRNA sequencing | PHR (pregnant hrHPV+) vs PN (pregnant HPV−) | Vaginal microbiome diversity and composition, community state types (CSTs), Lactobacillus abundance | Pregnancy and hrHPV infection both increased vaginal bacterial richness and diversity; reduced Lactobacillus abundance observed. hrHPV infection and pregnancy associated with higher prevalence of CST I (L. crispatus-dominant) and CST IV (anaerobic bacteria). Distinct bacterial taxa (Megasphaera, Sneathia, Prevotella, Gardnerella) enriched in hrHPV+ women; synergistic effects of pregnancy and hrHPV on vaginal microbiome suggested. |
| 17 | Łaniewski et al., 2018 | USA | Cross-sectional observational study | n = 100 women (non-Hispanic and Hispanic; reproductive age) | HPV infection and severity of cervical neoplasia; vaginal pH, microbiota (16S rRNA), immune mediator levels | HPV− controls vs HPV+ groups stratified by disease severity (HPV+ normal cytology, LSIL, HSIL, ICC) | Vaginal microbiome composition, cytokine and chemokine levels, vaginal pH, and association with cervical disease severity | Abnormal vaginal pH and depletion of Lactobacillus correlated with increased severity of cervical neoplasm. Sneathia spp. and other BV-associated taxa (Gardnerella, Prevotella, Atopobium) enriched in HPV+ and precancerous/cancerous groups. IL-36γ identified as key immune mediator linked to invasive cervical cancer. Distinct immune–microbial signatures associated with HPV persistence and cervical carcinogenesis. |
| 22 | Ferrera et al., 2023 | Morocco | Cross-sectional observational study | n = 438 women, age 18–86 | Detection of HPV, Chlamydia trachomatis (nested PCR), HPV genotyping via Sanger sequencing | HPV/CT co-infection vs single infection (HPV only or CT only) vs no infection; normal vs abnormal cytology | Prevalence of HPV, CT, co-infection; association with cervical cytological abnormalities; risk factors | HPV detected in 32.3%, CT in 17.7%, co-infection in 13.4%. Co-infection associated with higher risk of abnormal cytology (OR 3.18, 95% CI 0.96–9.21; p=0.040). Risk factors: STI history & marital status for CT; smoking for co-infection.. |
| 23 | Bellaminutti et al., 2014 | Italy | Cross-sectional study | 441 (305 (mean age 36 ± 10 years,85 mean age 28 ± 10 years, 51 mean age 37 ± 10 years) | Co-detection of HPV and Chlamydia trachomatis DNA | HPV− / CT− (no infection) vs HPV+ only vs CT+ only vs HPV+/CT+ co-detection | Prevalence of co-infection and type-specific HPV distribution | HPV and C. trachomatis were co-detected in 7.6% of participants. Co-infected women showed higher prevalence of high-risk HPV types. Results suggest that C. trachomatis may facilitate HPV persistence and increase cancer risk. |
| 24 | Samarawickrema et al., 2015 | Sri Lanka | Cross-sectional study | 483 women of age range 14–61, median 30 years) | Detection of Trichomonas vaginalis, Chlamydia trachomatis, Neisseria gonorrhoeae, and HPV | HPV+ vs HPV− (and/or STI-positive vs STI-negative) | Prevalence of STIs and co-infection patterns | HPV was the most prevalent infection (17.5%), followed by C. trachomatis and T. vaginalis. Co-infections were frequent, suggesting potential synergistic effects in cervical pathology development. |
| 25 | Garland et al., 2001 | Mongolia | Cross-sectional study | 110 women were studied (mean age 26.7 years) | Detection of Neisseria gonorrhoeae, Chlamydia trachomatis, Trichomonas vaginalis, and HPV | HPV+ vs HPV− (and/or STI-positive vs STI-negative) | Prevalence of HPV and co-infections | HPV detected in 27% of participants, C. trachomatis in 16%, and T. vaginalis in 10%. High prevalence of multiple infections suggests synergistic effects in cervical disease progression. |
| 27 | Shi W, Zhu H, Yuan L, Chen X, Huang X, Wang K, Li Z, 2022 | China | Prospective longitudinal cohort study | 85 Chinese woman with a single HR-HPV infection | Vaginal microbiota composition assessed by 16S rRNA sequencing | hrHPV clearance vs persistence (1-year follow-up) | Changes in vaginal microbiota diversity and dominant taxa; HPV clearance status | Higher abundance of Lactobacillus crispatus associated with HPV clearance; increased Gardnerella and Atopobium linked to persistence. Microbiota stability favored viral elimination. |
| 31 | Ou YC, Fu HC, Tseng CW, Wu CH, Tsai CC, Lin H, 2019 | Taiwan | Randomized, double-blind, placebo-controlled clinical trial | 121 women with genital HR-HPV infection were enrolled (62 in the study group and 59 in the control group) | Oral probiotics containing Lactobacillus rhamnosus and L. reuteri for 6 months | Probiotics vs placebo (hrHPV clearance) | HPV clearance rate, cytology improvement, vaginal microbiota composition | Probiotic group showed significantly higher hrHPV clearance and improved Pap smear cytology compared with placebo; restoration of Lactobacillus-dominant microbiota correlated with viral clearance. |
| 32 | Morales et al., 2022 | Brazil / Canada | Cross-sectional, nested within cervical cancer screening cohort | 609 reproductive-aged women. | Vaginal microbiome profiling via 16S rRNA sequencing | hrHPV-positive vs hrHPV-negative women | Association of microbial community types with hrHPV infection and cytological abnormalities | HPV-positive women had reduced Lactobacillus crispatus and increased Gardnerella, Atopobium, Prevotella. Community State Type IV strongly associated with hrHPV persistence and abnormal cytology. |
| 33 | Alaoui Sosse et al., 2023 | Morocco | Cross-sectional | 73 HPV positive cervical cancer samples | HPV–viral co-infection (EBV, HHV-8, HSV-2) | HPV co-infected vs HPV-only cervical cancer samples | Frequency of viral co-infection in cervical cancer tissues | High prevalence of HPV and EBV co-infection observed; viral synergy may promote oncogenic transformation through immune evasion and chronic inflammation mechanisms. |
