# Supplementary material for: Systems analysis of the HPV–microbiome–biofilm triad
Source: Front Cell Infect Microbiol. 2026 Mar 17;16:1767224. doi: 10.3389/fcimb.2026.1767224 (PMC13036498; doi:10.3389/fcimb.2026.1767224)
Supplement: Supplementary file 5 [file Table6.docx]

### Supplementary T****able** **S6**. **Summary of experimental and mechanistic studies on HPV–microbiome–host interactions****

| **№** | **Study (author, year)** | **Country** | **Design** | **Population / sample size (n) or experimental replication** | **Intervention / Exposure** | **Comparator** | **Outcomes measured** | **Key findings** |
| --- | --- | --- | --- | --- | --- | --- | --- | --- |
| **6** | **Dong J et al., 2023** | China | Experimental microbiological study | Bacterial biofilm model (E. faecalis, E. coli O157:H7, S. enteritidis); 4 biological × 3 technical replicates (12 readings); RNA-seq: 3 biological replicates | Coexistence of multiple bacterial species within biofilms | Single-species biofilms | Biofilm architecture, antimicrobial tolerance, interspecies interactions | Finds that bacterial coexistence modifies biofilm structure, enhances resistance to antimicrobials, and promotes cooperative survival |
| **7** | Wang et al., 2021 | USA | Experimental in vitro study | Human cell lines (hTERT-RPE-1; A549-HPV16 E6/E7); 3 independent biological replicates per experiment | Infection with Chlamydia trachomatis and/or high-risk HPV | Uninfected cells (control) | Centrosome number and amplification, multinucleation, cell cycle progression | C. trachomatis induced centrosome amplification in a higher proportion of cells than HPV; combined infection showed additive effects; centrosome amplification by Chlamydia correlated with multinucleation and required mitotic progression; HPV and Chlamydia affect centrosomes through distinct mechanisms, supporting Chlamydia as a co-factor for HPV in carcinogenesis |
| **8** | **Challagundla et al., 2023** | India | Experimental in vitro study | Cervical cancer cell lines (SiHa HPV16+; C-33A HPV−) + Chlamydia trachomatis; n/replicates not clearly reported | Co-infection with Chlamydia trachomatis and HPV E6/E7 oncogenes | HPV infection alone | Expression of HPV oncogenes, tumorigenesis, immune modulation | Demonstrates that Chlamydia trachomatis enhances HPV E6/E7-mediated oncogenic activity and immunosuppression, promoting tumor progression |
| 12 | Spardy et al., 2009 | USA | Experimental (cell-based study) | Primary human foreskin keratinocytes (HFKs), BJ fibroblasts, CaSki, C33A; n/replicates not reported | HPV-16 E7 expression | Control (non-E7-expressing cells) | Claspin degradation, checkpoint activation, DNA damage response | HPV-16 E7 increases degradation of claspin, impairing DNA damage checkpoint control and promoting genomic instability — a critical mechanism for HPV-induced carcinogenesis. |
| 13 | Fu et al., 2010 | USA / France (international collaboration) | Experimental in vitro comparative study | Cell line model (293T and C-33A) transfected with E6 proteins from multiple HPV types; 3 independent experiments | Expression of E6 proteins from 27 HPV types (alpha-HPV) in human cells; assessment of p53 degradation activity | Comparison between E6 proteins from different HPV types (oncogenic vs non-oncogenic; phylogenetic species groups) | p53 protein levels and degradation capacity measured by immunoblotting; correlation with HPV phylogeny | E6 from all Group 1 carcinogenic HPV types significantly degraded p53, but several non-oncogenic types (HPV53, HPV70, HPV71) showed similar activity. The ability of E6 to degrade p53 correlated more strongly with phylogeny than with oncogenic classification, indicating this function is evolutionarily conserved and not exclusively linked to carcinogenic potential. |
| 14 | Longworth et al., 2004 | USA | Experimental (molecular virology study) | **Primary human foreskin keratinocytes (HFKs) and COS cells; n/replicates not clearly reported** | HPV-31 E7 protein with intact vs. mutated zinc-finger motifs and HDAC-binding domains | Wild-type vs. mutant E7 constructs | Viral genome replication, transcription, and episome maintenance | Demonstrated that binding of histone deacetylases and integrity of zinc-finger-like motifs in HPV E7 are essential for viral life cycle completion and chromatin regulation. |
| 18 | Lebeau A et al., 2022 | Belgium | Experimental (multi-omics human study) | Retrospective cohort: N=6,117 women; human tissues: N=171 specimens | HPV infection status and mucosal antimicrobial peptide expression | HPV-negative women | Vaginal microbiome composition, mucosal peptide transcriptomics, proteomics | HPV infection down-regulated host mucosal peptides used by Lactobacilli as amino acid sources, shifting microbiota toward dysbiosis and reduced Lactobacillus abundance. |
| 19 | Castro et al., 2019 | Portugal | Experimental (in vitro polymicrobial model) | In vitro polymicrobial biofilm model; experiments repeated 3 times with technical replicates (up to 4 per assay). | G. vaginalis alone vs. mixed-species biofilms | Monospecies vs. polymicrobial biofilms | Biofilm biomass, structure, interspecies interactions | Demonstrated synergistic effects between G. vaginalis and other BV-associated species, resulting in denser, more resilient biofilms with increased tolerance to antimicrobials. |
| 20 | Castro et al., 2017 | Portugal / USA | Experimental (transcriptomic analysis, RNA-seq) | In vitro G. vaginalis biofilm vs planktonic cultures; RNA-seq sample size/biological replicates not explicitly stated (pooled biofilm material); qPCR validation performed with 3 biological replicates per condition. | Biofilm-forming G. vaginalis cells (72 h culture) | Gene expression profiles Planktonic G. vaginalis cells | Differential gene expression, virulence factors, biofilm formation genes | Biofilm cells showed up-regulation of genes involved in adhesion, antimicrobial resistance, and stress response compared with planktonic cells, suggesting biofilm-specific adaptations enhancing persistence. |
| 21 | Castro et al., 2020 | Portugal / Belgium | Experimental in vitro co-culture model | In vitro dual-species biofilm model (G. vaginalis + A. vaginae); assays repeated 3 times on separate days with technical replicates (2–4 per assay); PNA-FISH quantified across 20 randomly acquired fields per sample. | Co-culture of G. vaginalis and A. vaginae biofilms | Mono-culture biofilms of each species | Viability of A. vaginae, biofilm biomass, metabolic activity | G. vaginalis significantly increased A. vaginae viability and biofilm stability. Co-culture enhanced biofilm biomass and tolerance to antibiotics, suggesting a synergistic relationship contributing to bacterial vaginosis persistence. |
| 26 | Karim et al., 2013 | USA | Experimental in vitro study | Primary human keratinocytes (foreskin/vaginal; donor n not reported); seeding 7.5×10⁴–5×10⁵ cells/well; HPV16 infection MOI 100; lentiviral shRNA MOI 5. | HPV infection and expression of viral oncoproteins | Uninfected keratinocytes | Expression levels of UCHL1, interferon pathway markers, and immune signaling proteins | HPV upregulates deubiquitinase UCHL1, suppressing innate immune signaling and promoting immune evasion. Identifies a mechanism for viral persistence and immune escape. |
| 28 | **Li et al., 2020** | China | Experimental in vitro study | 10 BV patients; 10 clinical G. vaginalis isolates (+ ATCC 14018 control). | Antimicrobial susceptibility testing of G. vaginalis to metronidazole and clindamycin in planktonic vs. biofilm states | Planktonic vs. biofilm forms of G. vaginalis | Minimum inhibitory concentration (MIC), biofilm biomass, antibiotic tolerance | Biofilm-forming G. vaginalis showed significantly increased resistance to metronidazole and clindamycin compared to planktonic cells; standard treatment doses may be insufficient for biofilm-associated infections. |
| 29 | Rosca et al., 2022 | Portugal | Experimental in vitro biofilm model | In vitro triple-species BV biofilm model (G. vaginalis, F. vaginae, P. anaerobius); ≥3 independent experiments with technical duplicates (most assays); qPCR in triplicate | Co-culture biofilm exposed to metronidazole and clindamycin | Single-species vs. multi-species biofilm | Biofilm mass, metabolic activity, antibiotic tolerance | Multi-species BV biofilms exhibited higher antimicrobial tolerance than single-species biofilms; interspecies interactions enhance persistence and recurrence potential in BV. |
| 30 | Sabbatini et al., 2020 | Italy / France | Experimental in vitro study | In vitro G. vaginalis biofilm model (96-well plates); triplicate samples; 3–5 independent experiments | Co-incubation of G. vaginalis with probiotics Saccharomyces cerevisiae CNCM I-3856 and Lacticaseibacillus rhamnosus ATCC 53103 | G. vaginalis monoculture | Biofilm biomass, bacterial viability, adhesion inhibition | Both probiotic strains significantly reduced G. vaginalis biofilm formation and viability; S. cerevisiae CNCM I-3856 showed stronger anti-biofilm and anti-adhesion effects. |
